# Sugar beet extract rich in glycine betaine modulates oxidative defense system and key physiological characteristics of maize under water-deficit stress

**DOI:** 10.1371/journal.pone.0254906

**Published:** 2021-11-29

**Authors:** Sidra Shafiq, Nudrat Aisha Akram, Muhammad Ashraf, Mohammad S. AL-Harbi, Bassem N. Samra

**Affiliations:** 1 Department of Botany, Government College University, Faisalabad, Pakistan; 2 University of Agriculture, Faisalabad, Pakistan; 3 Department of Biology, College of Sciences, Taif University, Taif, Saudi Arabia; Institute for Biological Research, University of Belgrade, SERBIA

## Abstract

Now-a-days, plant-based extracts, as a cheap source of growth activators, are being widely used to treat plants grown under extreme climatic conditions. So, a trial was conducted to assess the response of two maize (*Zea mays* L.) varieties, Sadaf (drought tolerant) and Sultan (drought sensitive) to foliar-applied sugar beet extract (SBE) under varying water-deficit conditions. Different SBE (control, 1%, 2%, 3% & 4%) levels were used in this study, and plants were exposed to water-deficit [(75% and 60% of field capacity (FC)] and control (100% FC) conditions. It was observed that root and shoot dry weights (growth), total soluble proteins, RWC-relative water contents, total phenolics, chlorophyll pigments and leaf area per plant decreased under different water stress regimes. While, proline, malondialdehyde (MDA), RMP-relative membrane permeability, H_2_O_2_-hydrogen peroxide and the activities of antioxidant enzymes [CAT-catalase, POD-peroxidase and SOD-superoxide dismutase] were found to be improved in water stress affected maize plants. Exogenous application of varying levels of SBE ameliorated the negative effects of water-deficit stress by enhancing the growth attributes, photosynthetic pigments, RWC, proline, glycinebetaine (GB), activities of POD and CAT enzymes and levels of total phenolics, whereas it reduced the lipid peroxidation in both maize varieties under varying water stress levels. It was noted that 3% and 4% levels of SBE were more effective than the other levels used in enhancing the growth as well as other characteristics of the maize varieties. Overall, the sugar beet extract proved to be beneficial for improving growth and metabolism of maize plants exposed to water stress.

## Introduction

Water-deficit stress is one of the most crucial environmental cues for growth and yield outcomes of crops grown either in natural or agricultural systems, because an optimum amount of water is essential for the normal functioning of all metabolic activities taking place within the cells or tissues [1,2]. Water stress induces osmotic stress, overproduces reactive oxygen species (ROS), causes stomatal closure, perturbs carbohydrate assimilation, alters gas exchange characteristics and nutrients’ uptake. All individually or in combination are the major functions which alter the growth and yield production of major crops [3–7]. Owing to stressful conditions, the accumulation of ROS in the cellular organelles, e.g., mitochondria, chloroplasts, and peroxisomes results in membrane damage, ion leakage and inactivation of key enzymes [8–11].

Stress resistant plants can regulate their growth and development by improving the rates of photosynthesis, ion flux, respiration, carbohydrate metabolism, upregulating oxidative defense system, and enhancing the levels of plant growth promoters that usually undergo impairment under stressful environments [12,13]. It is now well evident that some metabolites including total phenolics, GB, carbohydrates and proline help sustain plant growth by improving drought tolerance and neutralizing ROS [14,15]. The scavenging network of ROS which includes enzymatic antioxidants such as catalase (CAT), peroxidase (POD), superoxide dismutase (SOD), and ascorbate peroxidase (APX), and non-enzymatic antioxidants including glutathione, tocopherols, ascorbate (non-enzymatic) antioxidants helps mediate the harmful effects of stressful factors in plants [16–20].

Plant based extracts are natural sources of organic and inorganic compounds that can be used exogenously to improve stress tolerance of plants [21]. Sugar beet (*Beta vulgaris* L.) extract is rich in flavonoids, phenolics, ascorbic acid, carotenoids and GB [22]. Exogenous application of SBE has been used to neutralize the negative influences of water stress conditions on plants [22,23]. Recently, foliar application of SBE has been shown to enhance growth, photosynthesis, antioxidants and yield of wheat plants subjected to water stress conditions [22]. Glycinebetaine is a well-known osmoprotectant that enables plants to sustain growth under stress conditions. It has multiple roles in the survival of different plants under non-stress and stress conditions [24,25]. Foliar application of glycinebetaine usually increases endogenous GB concentration in GB low- or non-accumulator plant species and improves growth and yield by counteracting the adverse effects of stress conditions [26,27]. It has been reported that GB mostly translocates to young tissues rather than older ones [26,28]. Earlier studies somehow prove the promotive effect of exogenous application of GB on yield, growth as well as different physio-biochemical attributes involved in stress tolerance of many crops such as wheat, sunflower, maize, tobacco, pea, etc. [29–33].

Maize (*Zea mays* L.) plant generally requires a high amount of water to complete its life cycle. However, when the crop is subjected to water deficit conditions, its growth and reproduction are adversely affected. Thus, for cultivating this crop in water deficit regions, some cost-effective and efficient measures must be undertaken. One of the key means of improving crop stress tolerance is the exogenous application of growth regulator substances, inorganic or organic in nature. As stated earlier, sugar beet extract has been reported to contain a significant amount of natural GB along with a variety of inorganic nutrients and organic substances [34,35]. In a previous study with okra, Habib, Ashraf [34] have shown that the sugar beet extract as a foliar spray ameliorated the negative effects of salinity on this potential vegetable crop. Thus, in the present study, we applied fresh extract of sugar beet to maize plants grown under water-deficit conditions and observed alterations occurring in different physiological and metabolic processes in maize plants.

## Materials and methods

To assess the antioxidative defense system in maize (*Zea mays* L.) plants, an experiment was carried-out under natural environment. So, seeds of selected maize varieties [36] viz. Sadaf (drought tolerant) and Sultan (drought sensitive) were soaked in water before germination. Then eight seeds were planted per plastic pot having 8 kg soil. The properties of sandy-loam experimental soil were as follows: EC, 2.09 dS m^-1^; pH, 7.45; P, 2.53 mg kg^-1^; K, 159 mg kg^-1^; organic matter, 0.92%; saturation, 35%; sand,48.7%; silt, 23.4%; clay, 27.4%; Zn, 0.46 mg kg^-1^; iron, 2.06 mg kg^-1^ and copper, 0.17 mg kg^-1^. Following one week of seed germination, seedlings were uniformly maintained to five seedlings per pot by hand thinning. Plants were subjected to different water stress (100%, 75% and 60% of FC) levels after two weeks of germination. Field capacity was calculated on the basis of moisture contents and the saturation percentage of the soil used. Common beet (*Beta vulgaris* L.) known as sugar beet was obtained from the local market of Faisalabad, Pakistan. Different concentrations (control, 1%, 2%, 3% & 4%) of SBE were applied as a foliar spray after two weeks of water deficit treatment. Half a liter of each solution concentration was prepared and 4% was used as a stock solution to prepare other concentrations. Tween-20 was mixed to each solution and an aliquot (10 mL) of the solution was applied per plant using a plastic sprayer. Two plants were harvested from each replicate after 14 days of exogenous treatment. The plant samples were washed with deionized water and growth parameters including root and shoot fresh and dry weights and leaf area per plant were determined. The following attributes were also measured on the harvested samples:

### Chlorophyll contents

A young top 3^rd^ leaf (500 mg) was extracted in 80% of acetone. To determine chlorophyll contents, optical densities of all extracts were observed using a spectrophotometer at 663 and 645 nm following Arnon [37].

### Relative water contents (RWC)

Fresh weights of young leaf samples were measured and dipped in water for an hour to note turgid weights. Then, the leaf samples were dried for appraising dry weights. The RWCs of young leaf samples were calculated according to Barrs and Weatherley [38].

### Relative membrane permeability (RMP)

Following the protocol of Yang, Rhodes [39], RMP was measured. A leaf (0.5 g) was taken and dipped in water to record EC_0_ using an EC meter. Then, EC_1_ was measured after keeping samples for 24 h at 4°C. Then, EC_2_ was noted after incubating the samples.

### Proline

Sulfo-salicylic acid (3%) was used to grind a fresh leaf (0.5 g), and proline contents were determined following the protocol of Bates, Waldren [40]. To the filtrate, glacial acetic acid (2 mL) and acid ninhydrin (2 mL) were added. The mixtures were kept in a water bath at 100°C for half an hour. After cooling, 4 mL toluene were added and shaken for 30 sec and absorbance recorded at 520 nm. For proline estimation, standard solutions of varying concentrations of proline (10–100 ppm) were used to draw a standard curve.

### Glycinebetaine

A fresh leaf (0.5 g) was homogenized with 0.5% toluene. After filtration, sulfuric acid (1 mL; 2 *N*) was added to 1 mL of the sample extract. Then, KI_3_ (0.2 mL) was mixed, cooled for 90 min and 1, 2 dichloroethane (5 mL) along with 2.8 mL of distilled water were added to the reacted sample. The absorbance of the lower layer was recorded at 365 nm Grieve and Grattan [41].

### Malondialdehyde (MDA)

Following Cakmak and Horst [42], the lipid peroxidation induced by water deficit conditions in the maize plants was determined. Fresh leaf (0.25 g) was homogenized with TCA (3 mL; 1%). To 1.0 mL of the filtrate, thiobarbituric acid (4 mL; 0.5% in 20% TCA) was added and incubated at 95°C for 50 min. The OD was observed at 532 and 600 nm.

### Hydrogen peroxide (H_2_O_2_)

A young leaf (0.25 g) was extracted in 0.1% of TCA (5 mL). To 0.5 mL leaf extract, 500 μL potassium phosphate buffer and 1 mL potassium iodide were mixed. The samples were vortexed and hydrogen peroxide contents measured at 390 nm Velikova, Yordanov [43].

### Total phenolics

A leaf (100 mg) was ground in 80% of acetone (5 mL) and the mixture was centrifuged. Then, 0.1 mL aliquot, 1 mL Folin-Ciocalteau’s reagent and 2 mL of deionized water were mixed. After shaking, 20% of sodium carbonate (5 mL) was added and maintained final volume up to 10 mL using distilled water. Total phenolics were measured at 750 nm [44].

### Ascorbic acid (AsA)

Following Mukherjee and Choudhuri [45], ascorbic acid contents in the maize leaf (0.25 g) were measured by extracting in 10 mL of TCA (6%). One drop of thiourea (10%; used 70% ethanol) along with 2% dinitrophenyl hydrazine (2 mL in 9 *N* H_2_SO_4_) was added to four mL of the sample aliquot. The solution was incubated for 15 min and cooled. The OD was measured at 530 nm after the addition of 80% H_2_SO_4_ (5 mL).

### Enzymatic antioxidants

Fresh leaf tissue (each 500 mg) was ground in 10 mL phosphate buffer having pH 7.8 and the extract was used for the determination of activities of antioxidant enzymes, i.e., CAT, SOD and POD. The activities of POD and CAT enzymes were measured following Chance and Maehly [46]. However, the activity of superoxide dismutase enzyme was determined according to the protocol of Giannopolitis and Ries [47].

#### Activity of SOD enzyme

In a cuvette, deionized water (0.4 mL), phosphate buffer (0.25 mL), triton-X (0.1 mL; 0.1%), L-methionine (0.1 mL; 13 mM), nitroblue tetrazolium (0.05 mL), riboflavin (50 μL of 1.3 μM) and enzyme extract (50 μL) were added. The OD of the samples was noted at 560 nm after 15 minutes.

#### Activity of POD enzyme

The activity of peroxidase (POD) enzyme was determined by preparing a reaction mixture (100 μL of 0.5% H_2_O_2_, 100 μL of 0.5% guaiacol, 1.8 mL phosphate buffer and 100 μL enzyme extract). The OD of this mixture was noted at 470 nm for 30 sec. time internal up to 3.0 minutes.

#### Activity of CAT enzyme

To 100 μL of the extract, H_2_O_2_ (1 mL of 5.9 mM) and phosphate buffer (50 mM; 1.9 mL) were added. The absorbance of each treated sample was noted at 240 nm for 180 seconds.

### Total soluble proteins

For this, the method of Bradford [48] was followed. To 100 μL aliquot of the sample, 2.0 mL of the Bradford reagent were mixed, and the OD was read at 590 nm.

### Statistical analysis

The obtained data were analyzed using a statistical package (Cohort software, Costat V6.303) to work out analysis of variance. LSD (least significance difference) test was used to compare the means of all treatments following Snedecor and Cochran [49].

## Results

**Fig 1:**
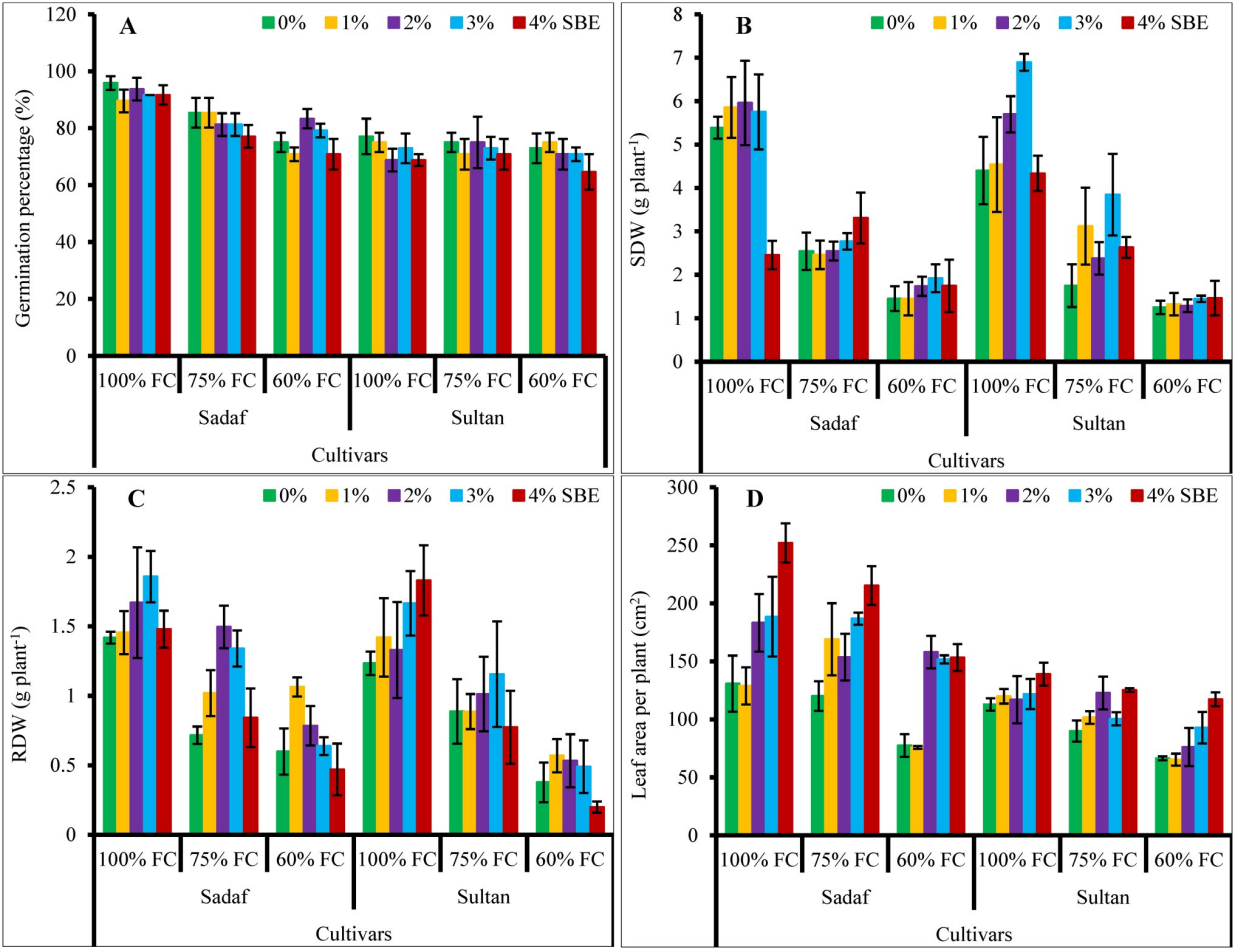
Effect of varying levels of sugar beet extract (SBE) on morphological attributes of maize plants subjected to water-deficit stress (*n* = 3; Mean ± S.E.).

Data for growth characteristics showed that shoot and root dry biomass decreased noticeably (*P* ≤ 0.001) under different water-deficit conditions. However, exogenously applied SBE at different levels were effective in enhancing the growth attributes of both maize varieties under water-deficit stress (Fig 1). SBE at the concentration of 3% was more effective in enhancing shoot fresh and dry weights, while 4% for leaf area of the water stressed plants. The response of both maize varieties to water stress conditions varied and var. Sadaf was better than var. Sultan at varying water-deficit regimes (Table 1).

**Table 1:** Three-way ANOVA of data for morphological and biochemical variables of water stressed plants of two maize varieties subjected to varying (1%, 2%, 3% & 4%) levels of sugar beet extract (SBE).

Source of variation	Df	Shoot dry weight	Root dry weight	Chl. a	Chl. b	Total Chl	Chl a/b ratio
Water stress (WS)	2	135. 5***	9.306***	2.185***	1.733***	6.994***	0.566**
SBE	4	4. 594**	0. 437*	0.403***	0.835***	2. 275***	0.144ns
Varieties (Var)	1	0.126ns	0.822*	4.137***	3.503***	15.25***	0.423*
WS x Var	2	0.442ns	0. 102ns	0.766***	1.241***	3. 957***	0.126ns
WS x SBE	8	3.573**	0. 235ns	0.141***	0.205**	0.426***	0.204*
Var x SBE	4	1.504ns	0.115ns	0.111**	0.098ns	0. 199ns	0.090ns
WS x SBE x Var	8	1.844ns	0. 084ns	0.186***	0.294***	0.695***	0.168*
Error	90	1.114	0.163	0.030	0.062	0.082	0.077
	Df	Leaf area	Relative Water Content	Relative Membrane Permeability	Proline	Glycinebetaine	Malondialdehyde
Water stress (WS)	2	23076.9***	1453.4***	4197.7***	2.097**	38.53ns	37.52*
Sugar beet extract (SBE)	4	16942.8***	1573.9***	1453. 5***	2.934***	437.6***	41. 33**
Varieties (Var)	1	80272.2***	1011.3***	403. 3ns	0.294ns	289.1***	21.67ns
WS x Var	2	1197.4ns	251.8***	3291.4***	0.285ns	2115.4***	0.495ns
WS x SBE	8	865.5ns	109.7***	149.1ns	0.496ns	46.96*	32. 73**
Var x SBE	4	4117.9**	68.47*	146.7ns	0.912ns	131.9***	18. 87ns
WS x SBE x Var	8	1526.3ns	31.83ns	129. 9ns	0.353ns	162.3***	0.811ns
Error	90	908.1	22.31	107.8	0.370	18.20	10.21
	df	Hydrogen peroxide	Total phenolics	Ascorbic acid	Catalase	Peroxidase	Superoxide dismutase
Water stress (WS)	2	269135.6***	41.78***	71.79***	44.07***	70.13***	481. 54***
Sugar beet extract (SBE)	4	40651.2ns	15.50***	20.22***	18.22**	28.09*	25.38ns
Varieties (Var)	1	277512.9***	12.96**	5.789ns	105. 8***	2546.2***	14.35ns
WS x Var	2	319739.4***	4.084*	19.45**	2.724ns	19.82ns	43. 58*
WS x SBE	8	118127.3***ns	0.795ns	1.407ns	2. 176ns	24.25**	5. 126ns
Var x SBE	4	26569.1ns	1.316ns	4.289ns	1.056ns	29.03*	8.392ns
WS x SBE x Var	8	87365. 02****	0.886ns	1.452ns	0.997ns	33.73***	4.764ns
Error	90	23833. 4	1.236	3.102	3. 865	8.844	12. 06

*, 0.05; **, 0.01; ***, 0.001 significance levels; ns, non significant.

Leaf area per plant was found to be significantly suppressed by water stress conditions. However, SBE spray under stress conditions remarkably (*P* ≤ 0.001, Table 1, Fig 1) enhanced the leaf area per plant in both maize varieties. Sadaf was better in leaf area than var. Sultan. The effects of maize varieties and that of the SBE treatment were significant.

**Fig 2:**
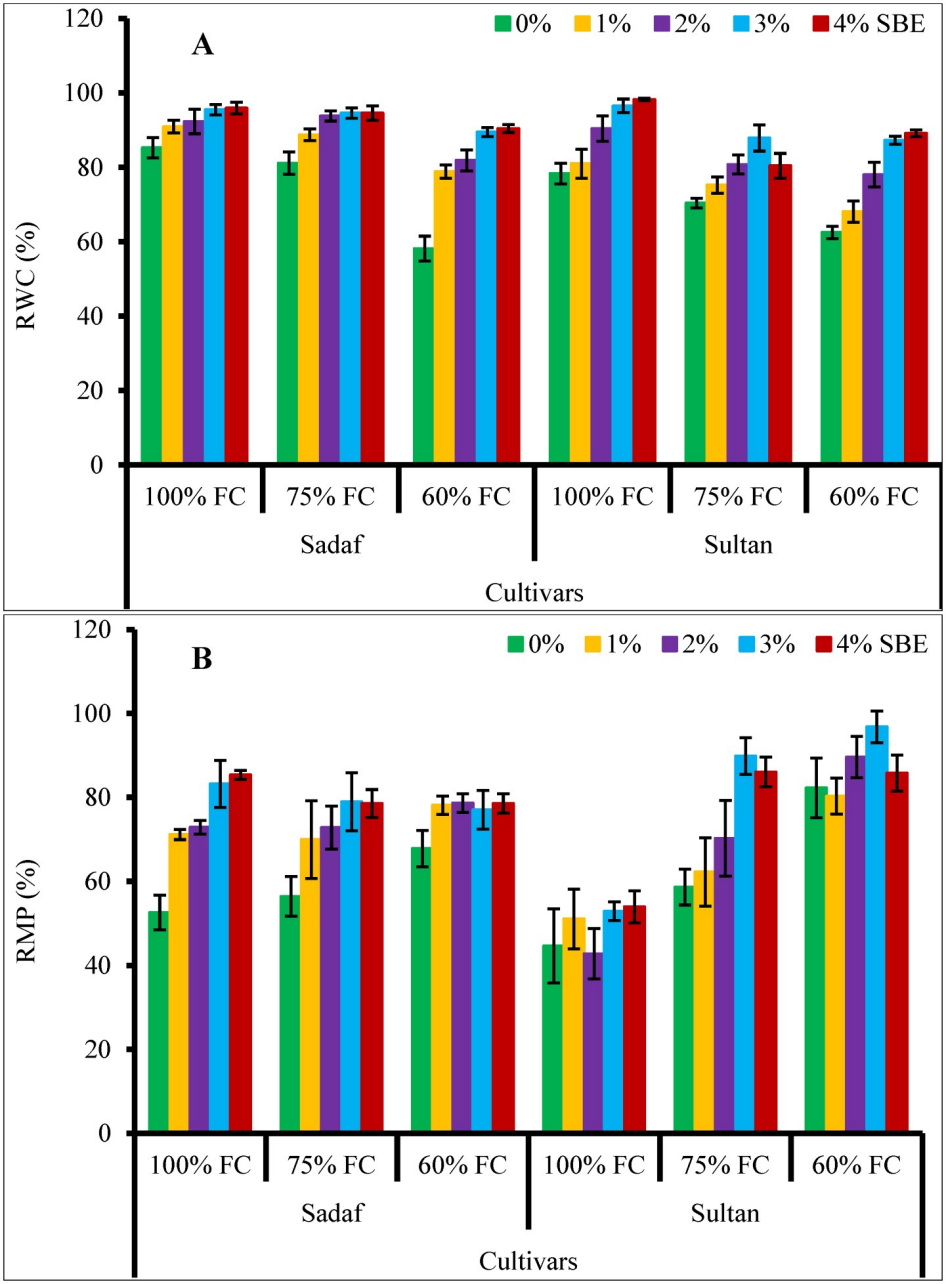
Effect of SBE on (A) RWC and (B) RMP of maize plants subjected to water-deficit stress (*n* = 3; Mean ± S.E.).

Relative water contents in the maize plants declined significantly (*P* ≤ 0.001) under water stress conditions. Foliar-applied SBE enhanced the RWC in all maize plants under water stress conditions (Fig 2, Table 1). Sadaf showed higher RWC compared to var. Sultan under varying watering regimes.

Water-deficit stress considerably (*P* ≤ 0.001, Table 1) increased the RMP in both maize varieties. While, SBE application at different (1% & 4%) concentrations reduced the RMP in var. Sultan under water stress (60% FC) conditions (Fig 2).

**Fig 3:**
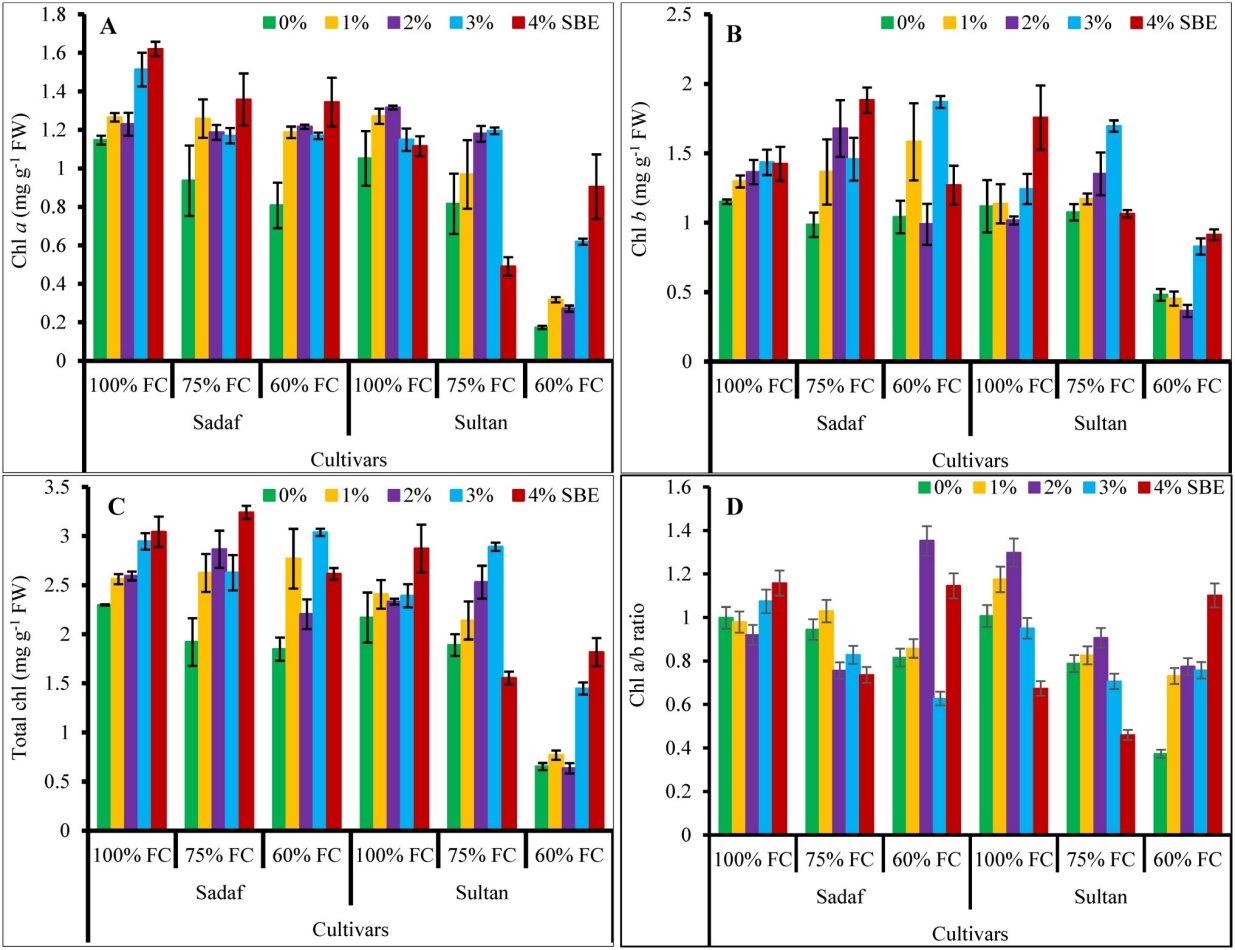
Restoration of chlorophyll pigments by supplementation of SBE of maize plants subjected to water-deficit stress (*n* = 3; Mean ± S.E.).

Chlorophyll pigments (*a*, *b*, total chlorophyll, *a/b* ratio) decreased significantly (*P* ≤ 0.001) under varying watering regimes. Foliar application of SBE at different concentrations improved the chlorophyll *a*, *b* and total chlorophyll under water-deficit stress (Table 1, Fig 3). While, no change was noted on chlorophyll *a/b* ratio. Both maize varieties showed consistency in all these pigment attributes (Fig 3). The interaction of SBE treatment, water stress and varieties was also significant.

**Fig 4:**
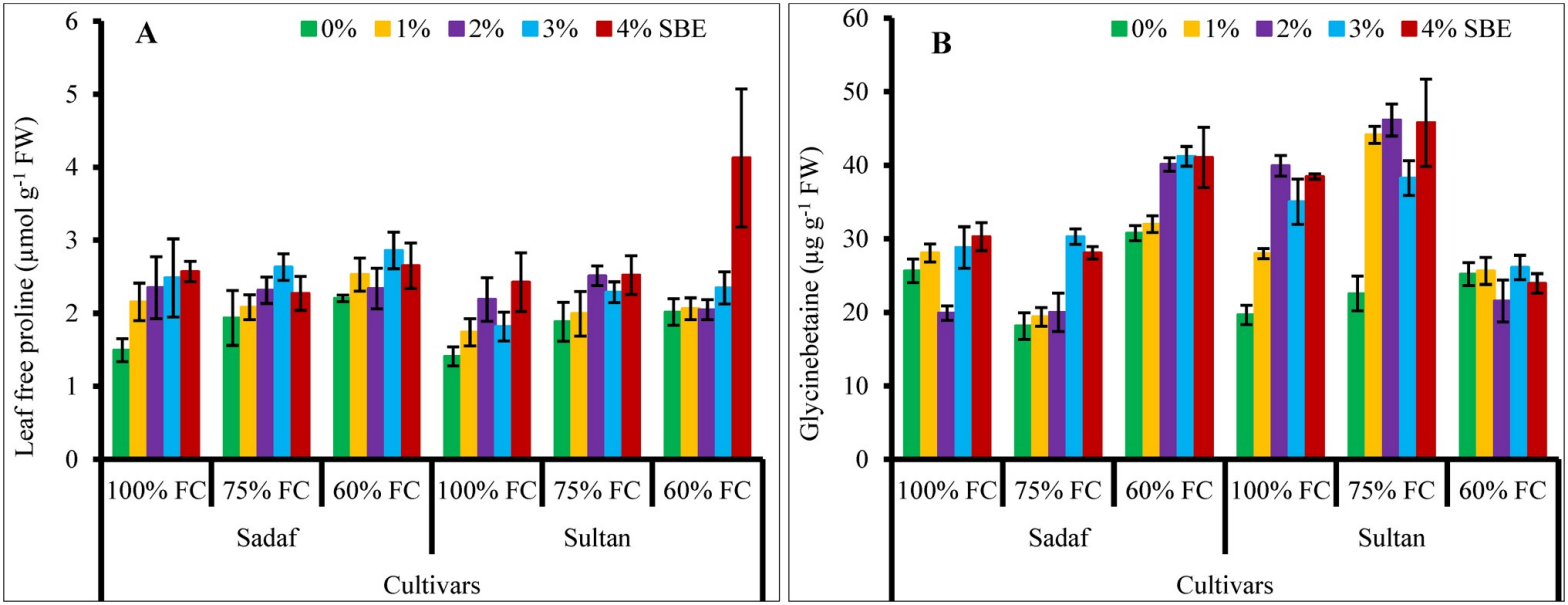
Application of SBE enhanced (A) leaf free proline and (B) glycinebetaine contents (GB) of maize plants subjected to water-deficit stress (*n* = 3; Mean ± S.E.).

Leaf free proline contents in both maize varieties increased significantly (*P* ≤ 0.01) under water stress. Foliage spray by SBE appreciably (*P* ≤ 0.001, Fig 4) enhanced the proline accumulation in both maize varieties under water stress conditions. Both varieties were similar in proline contents under water-deficit conditions.

No change in GB accumulation was noticed in maize plants under water stress. However, foliar-applied SBE noticeably (Table 1) enhanced the GB concentrations in both maize varieties under water stress. High GB contents were observed in var. Sadaf at 60% FC and in var. Sultan at 75% FC. The varieties, SBE treatment and water stress showed a significant interaction (Fig 4).

**Fig 5:**
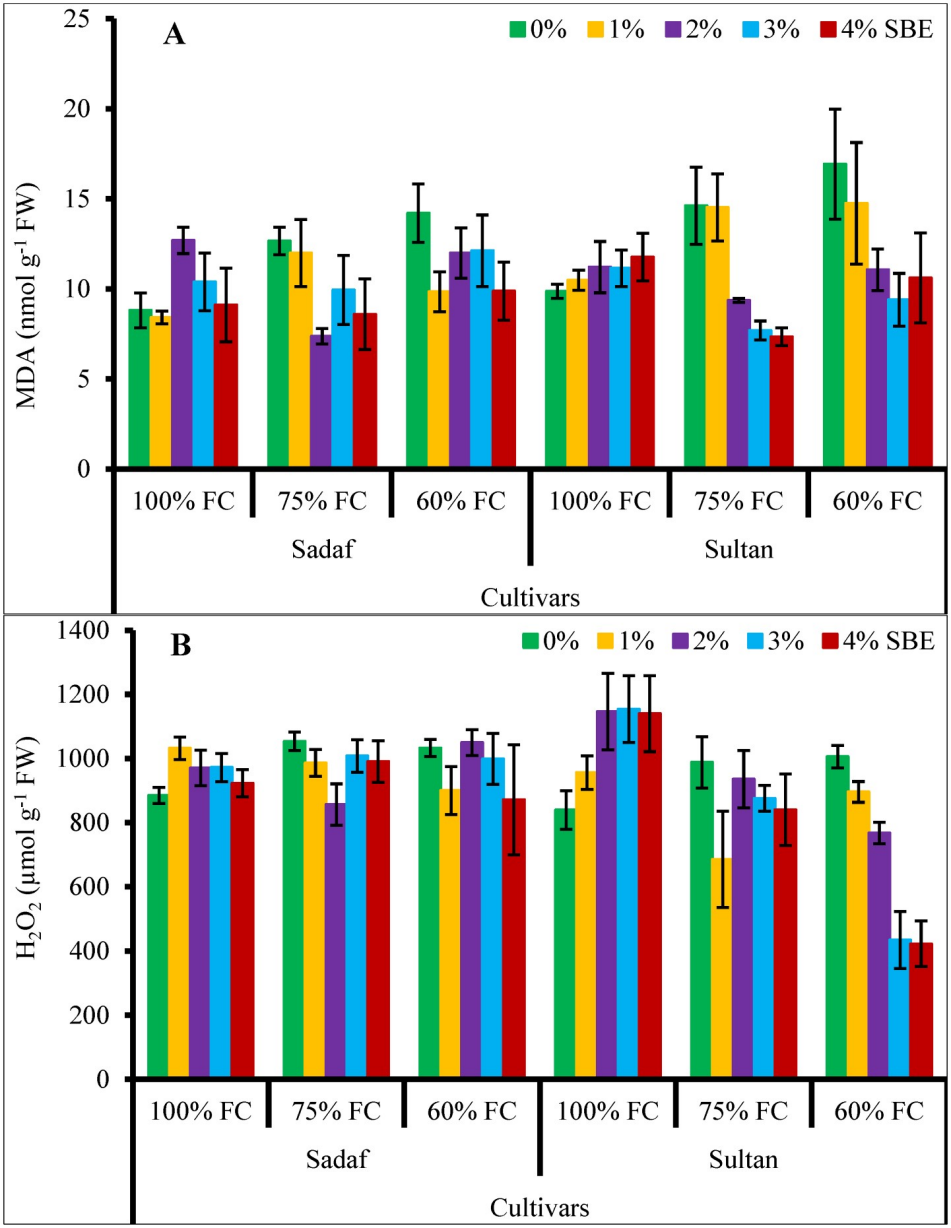
Sugar beet extract (SBE) regulates accumulation of (A) malondialdehyde (MDA) and (B) hydrogen peroxide (H_2_O_2_) content of maize plants subjected to water-deficit stress (*n* = 3; Mean ± S.E.).

Malondialdehyde (MDA) contents in the maize varieties were found to be improved (*P* ≤ 0.05) due to water-deficit stress. Exogenously applied SBE considerably (*P* ≤ 0.01, Fig 2) suppressed the MDA contents in both maize varieties under water stress conditions. Sultan had higher MDA contents than those of Sadaf under water-deficit stress (Table 1, Fig 5).

Hydrogen peroxide contents in the maize plants noticeably (*P* ≤ 0.001) enhanced under different watering regimes. Foliar application of SBE did not affect the H_2_O_2_ contents in all maize plants. Both maize varieties responded differentially under water deficit conditions and var. Sadaf accumulated relatively more H_2_O_2_ contents under water deficit conditions. The relationship among SBE application, water stress and varieties was significant (Table 1, Fig 5).

**Fig 6:**
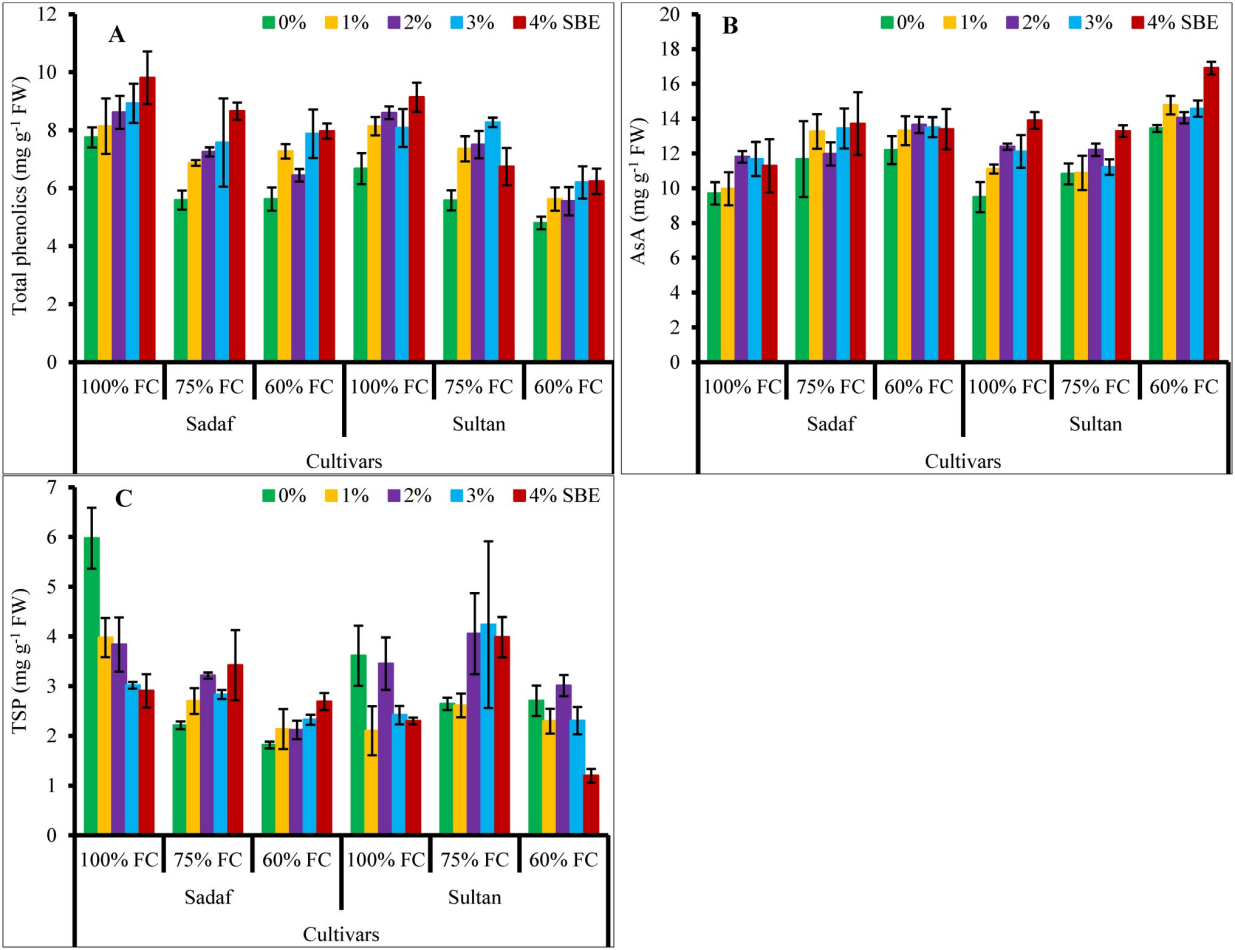
Effect of sugar beet extract (SBE) on (A) total phenolics, (B) ascorbic acid (AsA) and (C) total soluble proteins (TSP) of maize plants subjected to water-deficit stress (*n* = 3; Mean ± S.E.).

Water deficit conditions considerably (*P* ≤ 0.001) suppressed the total phenolics of the maize varieties. However, SBE applied as a foliar spray enhanced the total phenolics in the maize plants under water-deficit stress. Variety Sultan showed higher concentration of total phenolics under stress conditions compared with that in the other cultivar (Table 1, Fig 6).

Ascorbic acid (AsA) contents of the maize varieties significantly (*P* ≤ 0.001) improved under water-deficit stress as well as foliar application of SBE. The interface between varieties and water stress was significant (Fig 6). Both the varieties showed similar AsA contents in response to varying watering regimes.

Water-deficit stress considerably (*P* ≤ 0.001) decreased the total soluble proteins (TSP) in maize plants (Fig 6). While, SBE at different (1%, 2%, 3% & 4%) concentrations improved TSP in var. Sadaf at 75% and 60% FC. While in var. Sultan, SBE (2%, 3% & 4%) levels enhanced TSP at 75% FC, and 2% SBE level improved TSP at 60% FC. The interaction between water stress and varieties as well as between water stress and SBE was significant. The maize varieties did not differ significantly in total soluble proteins under water-deficit stress.

**Fig 7:**
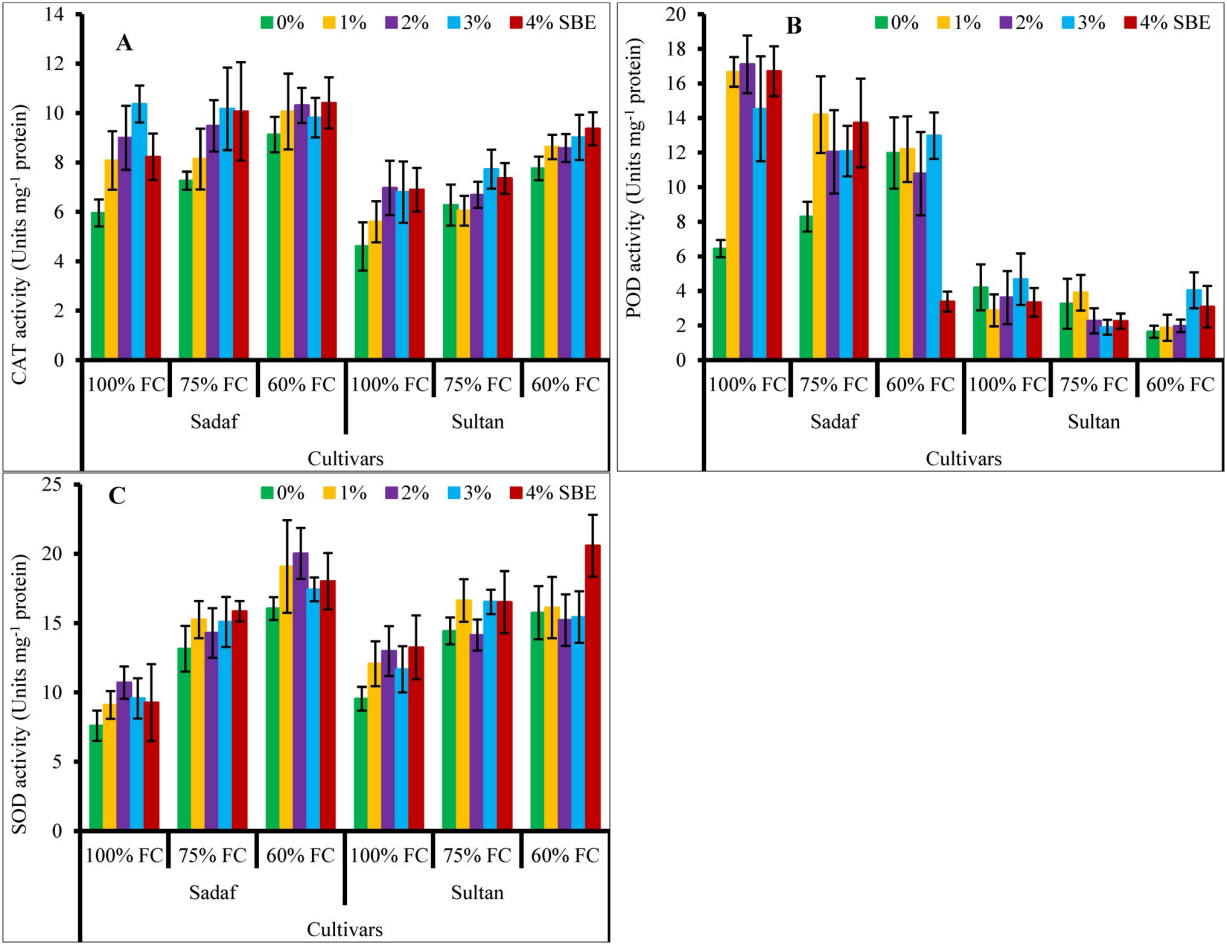
Supplementation of sugar beet extract (SBE) enhanced the activities of (A) CAT, (B) POD and (C) SOD enzymes of maize plants subjected to water-deficit stress (*n* = 3; Mean ± S.E.).

Varying watering regimes considerably (*P* ≤ 0.001) enhanced the activities of SOD, CAT and POD enzymes (Fig 7). Exogenous application of SBE also enhanced the activities of CAT and POD enzymes, while it did not affect the SOD activity under stress conditions. Variety Sadaf was better in CAT and POD activities than Sultan, whereas they both had a similar trend in SOD activity.

## Discussion

The present study was conducted to evaluate the effects of varying levels of sugar beet extract as a source of glycinebetaine on two maize varieties under water deficit conditions. Sugar beet is an excellent source of GB, carotenoids, AsA, flavonoids, phenolics, betalains etc. [22,23,34]. Overall, GB concentration in sugar beet is comparatively high i.e., 50 mM [47]. It can be applied to the foliage of plants for attaining improved stress tolerance of plants [22]. In the present study, growth characteristics (shoot and root dry weights) of all maize plants were suppressed under water-deficit stress conditions. However, these attributes were enhanced by foliar-applied SBE levels. Impairment in mitosis, and decreased turgor and water flow from xylem to other nearby cells may be the reasons for reduction in maize plants’ growth under water stress [1]. Recently, SBE was applied as a foliar spray to wheat plants under water stress [22]. It was illustrated that SBE ameliorated the drastic effects of water-deficit stress by increasing the growth, antioxidative defense system and nutrient homeostasis in wheat plants under stress conditions.

Chlorophyll pigments of the both maize varieties decreased under water deficit conditions in the current study. While, SBE treatment accelerated the photosynthetic pigments in all maize plants under stress conditions. Chlorophyll pigments decrease due to stomatal closure and reduction in leaf conductance, ultra-structural modifications to thylakoid lamellae, incomplete penetration of sunlight, and its use, inhibition in pigments of photosynthesis [50–52]. Another reason of reduced chlorophyll pigments could have been the production of ROS such as H_2_O_2,_ O_2_^-^, and ^1^O_2_ which might have led to lipid peroxidation and oxidative damages. A ROS, ^1^O_2_ is predominantly produced at PS II under stressful environment and it can lead to cell death under drought stress conditions [53]. Noman, Ali [22] reported an increase in chlorophyll pigments of wheat plants under water stress conditions which was found to be associated with enhanced plant growth. It was also reported that SBE acted as a bio-stimulant of plant growth under water-deficit stress conditions, since it also includes phenolics, flavonoids, ascorbic acid, etc. which can further reinforce the role of SBE as a growth promoter [21,22].

Cell growth is inhibited due to deficiency of water which leads to reduction in leaf development and leaf area, and hence overall reduced growth. As a result of reduced leaf area, transpiration rate decreases due to less uptake of water from soil. Leaf area determines the light interception and thus it is an important attribute to determine plant growth [54–56]. In the present investigation, leaf area was reduced by water stress conditions, but it was improved by foliar-applied SBE to the maize plants under water-deficit stress. Reduction in leaf area was also reported in *Sorghum* plants under drought stress by Yadav, Lakshmi [57]. It was reasoned that leaf area reduction might have been due to loss in turgor potential leading to less leaf expansion. In a recent study of pea plants, SBE along with other phytoextracts improved the leaf area of pea plants under salinity stress [35]. It might have been due to the presence of growth promoting compounds like, proline, GB, phenolics, flavonoids and tocopherols in the sugar beet extract [35,58].

Foliar applied SBE at different concentrations improved the RWCs of maize varieties which were reduced by the water-deficit conditions in the current study. Availability of water to soil reduces due to loss in transpiration, and an imbalanced water status leads to reduced plant RWC under drought stress conditions [59]. Reduction in relative water contents was found in many plants such as wheat [60], peanut [61], barley [62], pigeon pea [63], *Thymus citriodorus* [53], and canola [64] subjected to water deficit conditions. RWC can also be used as a potential indicator to explore better yielding genotypes which can balance cell turgor in response to water stress regimes [61,65].

Relative membrane permeability is considered as a measure of oxidative stress indicator in plant cells [66]. In the current investigation, RMP was found to be improved by water stress, whereas foliar application of SBE at different (1% and 4%) concentrations decreased the RMP in var. Sultan under 60% FC. Relatively drought sensitive cultivar, Sultan, showed high RMP under stress conditions. Similarly, water stress-induced elevation in RMP was also found in chickpea [66] and *Sesbania* [3] plants producing low biomass under arid conditions.

Osmoprotectants like proline, GB, soluble sugars and amino acids are soluble compounds which accumulate in plant species under stress conditions. These osmolytes help protect enzymes and macromolecules from ROS-induced damage under stress conditions [32,67,68]. In the present study with two maize varieties, proline and GB contents enhanced under water-deficit stress and foliar applied SBE. Likewise, Osman [32] reported that proline contents of pea plants increased which in turn reduced the lipid peroxidation and improved the growth of pea plants under water deficit conditions. Increase in GB and proline contents was also observed in wheat under water stress imposed by withholding of irrigation [69]. Proline and GB maintain stomatal conductance, photosynthesis, water balance and growth of plants through osmotic adjustment under stress conditions. These osmolytes not only help sustain plant life by osmoregulation [70], but also by regulation of enzymes’ activities and levels of proteins, scavenging of ROS and stabilization of membrane integrity [70,71].

Hydrogen peroxide is a highly reactive ROS and enzymes related to the Calvin cycle are more sensitive to it [72]. The MDA contents were improved due to production of ROS under stress conditions which can also be used as a stress indicator of lipid peroxidation [73]. In the maize plants, MDA and H_2_O_2_ contents increased under water stress conditions, while exogenously-applied SBE decreased the MDA contents, but in contrast, H_2_O_2_ contents remained unaffected under stress conditions. Recently, Avramova, AbdElgawad [74] reported that MDA and H_2_O_2_ contents increased in sensitive maize hybrids (EG3, EG4 and EG5) under varying (43% and 34% soil water contents) water regimes. Decrease in MDA and H_2_O_2_ contents by SBE might have been due to increase in the activities of SOD and POD enzymes under drought stress [22].

Total phenolics decreased in all maize plants under water stress conditions, while SBE applied at different concentrations enhanced it. Dissimilar to our findings, high accumulation of total phenolics was reported in *Aloe vera* [75] and rice [76] varieties under water stress.

Ascorbic acid is non-enzymatic antioxidant present in plant cells that can counteract ROS under stress conditions [77]. Ascorbic acid contents in the present study of maize plants increased due water stress and SBE treatment. It was examined in radish [78], tomato [79] and cauliflower [13] plants that AsA contents increased significantly under water stress. Ascorbic acid is involved in the defense of plant cell organelles from the oxidative damage caused due to ROS accumulation under stress conditions. It also acts as a cofactor of several enzymes, is involved in the biosynthesis of hormones, and restoration of antioxidants as well as it controls the division and expansion of cells [80–82].

Enzymatic antioxidants are involved in plant stress tolerance, cell redox balance as well as defense against oxidative damage [74,77,83–86]. In the current study, the activities of SOD, CAT and POD enzymes improved under water-deficit stress. Foliar spray of SBE at different concentrations also improved the activities of CAT and POD enzymes, but no change was observed in SOD activity. In a recent study on wheat plants, the activities of all these key enzymes were also reported to be improved by varying SBE (10%, 20%, 30%, 40% & 50%) levels under water stress (60% FC) conditions. It might have been due to the accumulation of osmoprotectants like GB that can act as an antioxidative compound to counteract ROS [22]. In another study, Shahid, Balal [35] observed that SBE with other phytoextract like that of moringa also enhanced the activities of enzymatic antioxidants in salt-stressed pea plants. These enzymes help eliminate the ROS, and a strong association between growth and antioxidants activities was found in this study.

## Conclusion

Overall, water stress (75% and 60% FC) decreased the growth attributes (shoot and root dry weights, leaf area), total soluble proteins, chlorophyll pigments, RWC, and total phenolics, whereas it enhanced proline, MDA, H_2_O_2_, RMP, AsA and the activities of CAT, SOD and POD enzymes. Foliar application of SBE enhanced all growth characteristics, RWC, chlorophyll pigments, proline, GB, total phenolics, AsA and the activities of the enzymatic antioxidants (CAT and POD). No change in total soluble proteins, H_2_O_2_ and SOD enzyme activity was observed due to the SBE application. However, SBE applied as 3% and 4% was more effective as compared to 1% and 2% SBE in counteracting the adversaries of water stress. Overall, var. Sadaf was better in growth, RWC and the activities of enzymatic antioxidants than var. Sultan.

## Supporting information

S1 Dataset (PDF)
