# Supplementary material for: Sugar beet extract rich in glycine betaine modulates oxidative defense system and key physiological characteristics of maize under water-deficit stress
Source: PLoS One. 2021 Nov 29;16(11):e0254906. doi: 10.1371/journal.pone.0254906 (PMC8629294; doi:10.1371/journal.pone.0254906)
Supplement: S1 Dataset — (PDF) [file pone.0254906.s001.pdf]

No.	Drought	Cultivars	Treatment	Replicates	SFW / plant
1	1	1	1	1	32.25
2	1	1	1	2	32.8
3	1	1	1	3	28.45
4	1	1	1	4	34.45
5	1	1	2	1	24.505
6	1	1	2	2	32.605
7	1	1	2	3	33.01
8	1	1	2	4	34.15
9	1	1	3	1	17.525
10	1	1	3	2	25.555
11	1	1	3	3	39.3
12	1	1	3	4	31
13	1	1	4	1	34.6
14	1	1	4	2	27.575
15	1	1	4	3	25.53
16	1	1	4	4	26.95
17	1	1	5	1	20.675
18	1	1	5	2	24.155
19	1	1	5	3	18.06
20	1	1	5	4	19.63
21	2	1	1	1	19.26
22	2	1	1	2	25.65
23	2	1	1	3	28.05
24	2	1	1	4	20.05
25	2	1	2	1	20.06
26	2	1	2	2	21.06
27	2	1	2	3	13.155
28	2	1	2	4	14.155
29	2	1	3	1	21.56
30	2	1	3	2	18.505
31	2	1	3	3	21.56
32	2	1	3	4	21.065
33	2	1	4	1	23.15
34	2	1	4	2	21.25
35	2	1	4	3	18.4
36	2	1	4	4	24.65
37	2	1	5	1	21.4
38	2	1	5	2	29.06
39	2	1	5	3	26.18
40	2	1	5	4	22.805
41	3	1	1	1	12.9
42	3	1	1	2	23.16
43	3	1	1	3	20.06
44	3	1	1	4	14.17
45	3	1	2	1	13.17

Individual level dataset
Shoot Fresh weight (S[illegible]

Cultivars **Sadaf**

Sultan

46	3	1	2	2	13.4
47	3	1	2	3	15.06
48	3	1	2	4	17.8
49	3	1	3	1	17.08
50	3	1	3	2	15.23
51	3	1	3	3	18.225
52	3	1	3	4	19.08
53	3	1	4	1	18.225
54	3	1	4	2	16.25
55	3	1	4	3	21.3
56	3	1	4	4	19.6
57	3	1	5	1	15.23
58	3	1	5	2	12.65
59	3	1	5	3	15.6
60	3	1	5	4	21.45
61	1	2	1	1	19.3
62	1	2	1	2	24.325
63	1	2	1	3	26.35
64	1	2	1	4	24.78
65	1	2	2	1	29.805
66	1	2	2	2	24.305
67	1	2	2	3	23.65
68	1	2	2	4	24.65
69	1	2	3	1	21.05
70	1	2	3	2	18.2
71	1	2	3	3	26.17
72	1	2	3	4	33.695
73	1	2	4	1	35.05
74	1	2	4	2	36.455
75	1	2	4	3	36.015
76	1	2	4	4	35.06
77	1	2	5	1	25.66
78	1	2	5	2	22.835
79	1	2	5	3	26.23
80	1	2	5	4	22.685
81	2	2	1	1	14.455
82	2	2	1	2	15.615
83	2	2	1	3	14.65
84	2	2	1	4	24.3
85	2	2	2	1	23.4
86	2	2	2	2	25.835
87	2	2	2	3	18.25
88	2	2	2	4	16.2
89	2	2	3	1	17.34
90	2	2	3	2	19.73
91	2	2	3	3	20.905

92	2	2	3	4	14.23
93	2	2	4	1	28.15
94	2	2	4	2	22.69
95	2	2	4	3	17.73
96	2	2	4	4	23.95
97	2	2	5	1	24.18
98	2	2	5	2	17.1
99	2	2	5	3	19.3
100	2	2	5	4	20.15
101	3	2	1	1	16.25
102	3	2	1	2	14.3
103	3	2	1	3	13.8
104	3	2	1	4	14.65
105	3	2	2	1	12.45
106	3	2	2	2	14.75
107	3	2	2	3	15.225
108	3	2	2	4	12.45
109	3	2	3	1	14.15
110	3	2	3	2	13.15
111	3	2	3	3	15.06
112	3	2	3	4	15.28
113	3	2	4	1	16.3
114	3	2	4	2	14.85
115	3	2	4	3	13.45
116	3	2	4	4	14.2
117	3	2	5	1	12.45
118	3	2	5	2	11.95
119	3	2	5	3	11.32
120	3	2	5	4	15.9

Individual level dataset

SDW

Shoot dry weight (SDW

No.	Drought	Cultivars	Treatment	Replicates	SDW/ plant
1	1	1	1	1	5.535
2	1	1	1	2	5.57
3	1	1	1	3	4.655
4	1	1	1	4	5.795
5	1	1	2	1	3.94
6	1	1	2	2	5.81
7	1	1	2	3	6.445
8	1	1	2	4	7.23
9	1	1	3	1	6.1
10	1	1	3	2	4.075

11	1	1	3	3	8.6		
12	1	1	3	4	5.07		
13	1	1	4	1	8.275		
14	1	1	4	2	5.24		
15	1	1	4	3	4.34		
16	1	1	4	4	5.16		
17	1	1	5	1	4.505		
18	1	1	5	2	6.725		
19	1	1	5	3	4.53	**Cultivars**	**Sadaf**
20	1	1	5	4	6.06		
21	2	1	1	1	1.28		
22	2	1	1	2	2.96		**Sultan**
23	2	1	1	3	2.75		
24	2	1	1	4	3.18		
25	2	1	2	1	3.19		
26	2	1	2	2	2.835		
27	2	1	2	3	1.925		
28	2	1	2	4	1.885		
29	2	1	3	1	2.75		
30	2	1	3	2	1.94		
31	2	1	3	3	2.565		
32	2	1	3	4	2.935		
33	2	1	4	1	3.315		
34	2	1	4	2	2.59		
35	2	1	4	3	2.44		
36	2	1	4	4	2.73		
37	2	1	5	1	2.54		
38	2	1	5	2	4.43		
39	2	1	5	3	2.085		
40	2	1	5	4	4.18		
41	3	1	1	1	1.295		
42	3	1	1	2	1.05		
43	3	1	1	3	2.29		
44	3	1	1	4	1.17		
45	3	1	2	1	1.915		
46	3	1	2	2	0.46		
47	3	1	2	3	2.155		
48	3	1	2	4	1.265		
49	3	1	3	1	1.61		
50	3	1	3	2	1.28		
51	3	1	3	3	1.72		
52	3	1	3	4	2.33		
53	3	1	4	1	1.33		
54	3	1	4	2	1.41		
55	3	1	4	3	2.57		
56	3	1	4	4	2.365		

57	3	1	5	1	1.15
58	3	1	5	2	0.96
59	3	1	5	3	1.34
60	3	1	5	4	3.535
61	1	2	1	1	2.75
62	1	2	1	2	4.01
63	1	2	1	3	6.485
64	1	2	1	4	4.355
65	1	2	2	1	7.775
66	1	2	2	2	3.535
67	1	2	2	3	3.06
68	1	2	2	4	3.78
69	1	2	3	1	4.68
70	1	2	3	2	5.385
71	1	2	3	3	6.56
72	1	2	3	4	6.16
73	1	2	4	1	5.015
74	1	2	4	2	7.15
75	1	2	4	3	5.1
76	1	2	4	4	6.315
77	1	2	5	1	4.305
78	1	2	5	2	5.425
79	1	2	5	3	4.155
80	1	2	5	4	3.455
81	2	2	1	1	0.875
82	2	2	1	2	1.97
83	2	2	1	3	1.115
84	2	2	1	4	3.04
85	2	2	2	1	3.67
86	2	2	2	2	3.36
87	2	2	2	3	2.04
88	2	2	2	4	1.405
89	2	2	3	1	2.17
90	2	2	3	2	2.96
91	2	2	3	3	2.98
92	2	2	3	4	1.41
93	2	2	4	1	3.26
94	2	2	4	2	2.15
95	2	2	4	3	2.79
96	2	2	4	4	3.19
97	2	2	5	1	3.155
98	2	2	5	2	1.995
99	2	2	5	3	2.72
100	2	2	5	4	2.655
101	3	2	1	1	1.64
102	3	2	1	2	0.96

103	3	2	1	3	1.045
104	3	2	1	4	1.365
105	3	2	2	1	0.575
106	3	2	2	2	1.525
107	3	2	2	3	1.47
108	3	2	2	4	1.725
109	3	2	3	1	0.85
110	3	2	3	2	1.456
111	3	2	3	3	1.395
112	3	2	3	4	1.455
113	3	2	4	1	1.26
114	3	2	4	2	1.415
115	3	2	4	3	1.56
116	3	2	4	4	1.56
117	3	2	5	1	2.13
118	3	2	5	2	0.675
119	3	2	5	3	2.16
120	3	2	5	4	0.89

Individual level dataset captions Table 3

Chlorophyll content

Chlorophyll content under SBE and water deficit stress

No.	Drought	Cultivars	Treatment	Replicates	ak2*0.05 (Chl a)		
1	1	1	1	1	1.20505		
2	1	1	1	2	1.132938		
3	1	1	1	3	1.095319		
4	1	1	1	4	1.154497		
5	1	1	2	1	1.301996		
6	1	1	2	2	1.304686		
7	1	1	2	3	1.237036		
8	1	1	2	4	1.217175		
9	1	1	3	1	1.306838		
10	1	1	3	2	1.215256		
11	1	1	3	3	1.328582		
12	1	1	3	4	1.067032		
13	1	1	4	1	1.255325		
14	1	1	4	2	1.326991		
15	1	1	4	3	1.420254		
16	1	1	4	4	1.548162		
17	1	1	5	1	1.050924		
18	1	1	5	2	1.326118		
19	1	1	5	3	1.58348	**Cultivars**	**Sadaf**
20	1	1	5	4	1.017021		
21	2	1	1	1	0.634178		
22	2	1	1	2	0.665386		**Sultan**
23	2	1	1	3	1.416512		

24	2	1	1	4	1.027377
25	2	1	2	1	1.209595
26	2	1	2	2	1.501078
27	2	1	2	3	1.305039
28	2	1	2	4	1.019134
29	2	1	3	1	1.22439
30	2	1	3	2	1.178282
31	2	1	3	3	1.082425
32	2	1	3	4	1.263021
33	2	1	4	1	1.192135
34	2	1	4	2	1.265416
35	2	1	4	3	1.147906
36	2	1	4	4	1.074216
37	2	1	5	1	1.325165
38	2	1	5	2	1.538809
39	2	1	5	3	1.577818
40	2	1	5	4	0.989616
41	3	1	1	1	0.557379
42	3	1	1	2	0.783508
43	3	1	1	3	0.761128
44	3	1	1	4	1.127483
45	3	1	2	1	1.108125
46	3	1	2	2	1.236752
47	3	1	2	3	1.173843
48	3	1	2	4	1.230392
49	3	1	3	1	1.202215
50	3	1	3	2	1.197376
51	3	1	3	3	1.217851
52	3	1	3	4	1.246377
53	3	1	4	1	1.167006
54	3	1	4	2	1.215906
55	3	1	4	3	1.137007
56	3	1	4	4	1.156222
57	3	1	5	1	1.106127
58	3	1	5	2	1.179558
59	3	1	5	3	1.662368
60	3	1	5	4	1.426877
61	1	2	1	1	1.280088
62	1	2	1	2	0.648622
63	1	2	1	3	1.061207
64	1	2	1	4	1.215226
65	1	2	2	1	1.276576
66	1	2	2	2	1.28056
67	1	2	2	3	1.359835
68	1	2	2	4	1.165838
69	1	2	3	1	1.322171

70	1	2	3	2	1.328797		
71	1	2	3	3	1.326021		
72	1	2	3	4	1.286567		
73	1	2	4	1	1.275308		
74	1	2	4	2	1.194897		
75	1	2	4	3	1.12425		
76	1	2	4	4	1.001392		
77	1	2	5	1	1.234208		
78	1	2	5	2	1.019868		
79	1	2	5	3	1.170615		
80	1	2	5	4	1.036377		
81	2	2	1	1	0.664896		
82	2	2	1	2	1.063043		
83	2	2	1	3	0.44625		
84	2	2	1	4	1.08931		
85	2	2	2	1	1.246448		
86	2	2	2	2	0.679265		
87	2	2	2	3	0.641057		
88	2	2	2	4	1.305846		
89	2	2	3	1	1.264548		
90	2	2	3	2	1.232406		
91	2	2	3	3	1.127143		
92	2	2	3	4	1.094083		
93	2	2	4	1	1.241145		
94	2	2	4	2	1.165243		
95	2	2	4	3	1.171295		
96	2	2	4	4	1.203262		
97	2	2	5	1	1.469258		
98	2	2	5	2	1.369458		
99	2	2	5	3	0.526485		
100	2	2	5	4	0.96256		
101	3	2	1	1	0.196854		
102	3	2	1	2	0.267774		
103	3	2	1	3	0.258649		
104	3	2	1	4	0.168953		
105	3	2	2	1	0.296458		
106	3	2	2	2	0.35552		
107	3	2	2	3	0.308599		
108	3	2	2	4	0.308599		
109	3	2	3	1	0.268952	0.580316	0.290158
110	3	2	3	2	0.234569		
111	3	2	3	3	0.312655		
112	3	2	3	4	0.267661		
113	3	2	4	1	0.333542		
114	3	2	4	2	0.235678		
115	3	2	4	3	0.569245		

116	3	2	4	4	0.336216
117	3	2	5	1	0.52286
118	3	2	5	2	0.40148
119	3	2	5	3	0.326848
120	3	2	5	4	0.401458

captions Table 1

FW) under SBE and water deficit stress

	CV1							
	100%FC					75% FC		
	0%	1%	2%	3%	4%	0%	1%	2%
	32.25	24.505	17.525	34.6	20.675	19.26	20.06	21.56
	32.8	32.605	25.555	27.575	24.155	25.65	21.06	18.505
	28.45	33.01	39.3	25.53	18.06	28.05	13.155	21.56
	34.45	34.15	31	26.95	19.63	20.05	14.155	21.065
Mean	31.9875	31.0675	28.345	28.66375	20.63	23.2525	17.1075	20.6725
SD	2.536853	4.423627	9.163652	4.048941	2.584089	4.28019	4.028193	1.46372
SE	1.268427	2.211813	4.581826	2.02447	1.292045	2.140095	2.014097	0.73186

	**0%**	**1%**	**2%**	**3%**	**4% SBE**
**100% FC**	31.9875	31.0675	28.345	28.66375	20.63
**75% FC**	23.2525	17.1075	20.6725	21.8625	24.86125
**60% FC**	17.5725	14.8575	17.40375	18.84375	16.2325
**100% FC**	23.68875	25.6025	24.77875	35.645	24.3525
**75% FC**	17.255	20.92125	18.05125	23.13	20.1825
**60% FC**	14.75	13.71875	14.41	14.7	12.905

captions Table 2

V) under SBE and water deficit stress

	CV1							
	100%FC					75% FC		
	0%	1%	2%	3%	4%	0%	1%	2%
	5.535	3.94	6.1	8.275	4.505	1.28	3.19	2.75
	5.57	5.81	4.075	5.24	6.725	2.96	2.835	1.94
	4.655	6.445	8.6	4.34	4.53	2.75	1.925	2.565
	5.795	7.23	5.07	5.16	6.06	3.18	1.885	2.935
Mean	5.38875	5.85625	5.96125	5.75375	5.455	2.5425	2.45875	2.5475
SD	0.50255	1.403326	1.943752	1.729342	1.116102	0.859782	0.655838	0.432252

SE	0.251275	0.701663	0.971876	0.864671	0.558051	0.429891	0.327919	0.216126

	**0%**	**1%**	**2%**	**3%**	**4% SBE**
**100% FC**	5.38875	5.85625	5.96125	5.75375	5.455
**75% FC**	2.5425	2.45875	2.5475	2.76875	3.30875
**60% FC**	1.45125	1.44875	1.735	1.91875	1.74625
**100% FC**	4.4	4.5375	5.69625	5.895	4.335
**75% FC**	1.75	2.61875	2.38	2.8475	2.63125
**60% FC**	1.2525	1.32375	1.289	1.44875	1.46375

	CV1							
	100%FC					75% FC		
	0%	1%	2%	3%	4%	0%	1%	2%
	1.20505	1.301996	1.306838	1.255325	1.050924	0.634178	1.209595	1.22439
	1.132938	1.304686	1.215256	1.326991	1.326118	0.665386	1.501078	1.178282
	1.095319	1.237036	1.328582	1.420254	1.58348	1.416512	1.305039	1.082425
	1.154497	1.217175	1.067032	1.548162	1.017021	1.027377	1.019134	1.263021
Mean	1.146951	1.265223	1.229427	1.387683	1.244386	0.935863	1.258711	1.187029
SD	0.045806	0.044769	0.11888	0.126513	0.26507	0.366774	0.200581	0.077865
SE	0.022903	0.022384	0.05944	0.063256	0.132535	0.183387	0.10029	0.038933

	**0%**	**1%**	**2%**	**3%**	**4% SBE**
**100% FC**	1.146951	1.265223	1.229427	1.387683	1.244386
**75% FC**	0.935863	1.258711	1.187029	1.169918	1.357852
**60% FC**	0.807374	1.187278	1.215955	1.169035	1.343732
**100% FC**	1.051285	1.270702	1.315889	1.148962	1.115267
**75% FC**	0.815875	0.968154	1.179545	1.195236	1.08194

**60% FC**	0.223057	0.317294	0.270959	0.36867	0.413161

							CV2	
		60% FC					100%FC	
3%	4%	0%	1%	2%	3%	4%	0%	1%
23.15	21.4	12.9	13.17	17.08	18.225	15.23	19.3	29.805
21.25	29.06	23.16	13.4	15.23	16.25	12.65	24.325	24.305
18.4	26.18	20.06	15.06	18.225	21.3	15.6	26.35	23.65
24.65	22.805	14.17	17.8	19.08	19.6	21.45	24.78	24.65
21.8625	24.86125	17.5725	14.8575	17.40375	18.84375	16.2325	23.68875	25.6025
2.695173	3.443691	4.858569	2.134735	1.664759	2.138182	3.717601	3.051722	2.832197
1.347587	1.721845	2.429284	1.067367	0.83238	1.069091	1.858801	1.525861	1.416099

		**0%**	**1%**	**2%**	**3%**	**4%**
**Cv1**	**100% FC**	1.268427	2.211813	4.581826	2.02447	1.292045
	**75% FC**	2.140095	2.014097	0.73186	1.347587	1.721845
	**60% FC**	2.429284	1.067367	0.83238	1.069091	1.858801
**Cv2**	**100% FC**	1.525861	1.416099	3.398757	0.352284	0.927269
	**75% FC**	2.361985	2.230749	1.473938	2.145274	1.479349
	**60% FC**	0.529544	0.738902	0.486021	0.605186	1.024748

							CV2	
		60% FC					100%FC	
3%	4%	0%	1%	2%	3%	4%	0%	1%
3.315	2.54	1.295	1.915	1.61	1.33	1.15	2.75	7.775
2.59	4.43	1.05	0.46	1.28	1.41	0.96	4.01	3.535
2.44	2.085	2.29	2.155	1.72	2.57	1.34	6.485	3.06
2.73	4.18	1.17	1.265	2.33	2.365	3.535	4.355	3.78
2.76875	3.30875	1.45125	1.44875	1.735	1.91875	1.74625	4.4	4.5375
0.382935	1.169732	0.568043	0.758852	0.43852	0.639979	1.202549	1.551757	2.178931

0.191468	0.584866	0.284022	0.379426	0.21926	0.319989	0.601274	0.775879	1.089466

		0%	1%	2%	3%	4%
**Cv1**	**100% FC**	0.251275	0.701663	0.971876	0.864671	0.558051
	**75% FC**	0.429891	0.327919	0.216126	0.191468	0.584866
	**60% FC**	0.284022	0.379426	0.21926	0.319989	0.601274
**Cv2**	**100% FC**	0.775879	1.089466	0.41741	0.512986	0.407819
	**75% FC**	0.490013	0.537178	0.374322	0.254505	0.239369
	**60% FC**	0.155838	0.255525	0.147027	0.0716	0.395808

							CV2	
		60% FC					100%FC	
3%	4%	0%	1%	2%	3%	4%	0%	1%
1.192135	1.325165	0.557379	1.108125	1.202215	1.167006	1.106127	1.280088	1.276576
1.265416	1.538809	0.783508	1.236752	1.197376	1.215906	1.179558	0.648622	1.28056
1.147906	1.577818	0.761128	1.173843	1.217851	1.137007	1.662368	1.061207	1.359835
1.074216	0.989616	1.127483	1.230392	1.246377	1.156222	1.426877	1.215226	1.165838
1.169918	1.357852	0.807374	1.187278	1.215955	1.169035	1.343732	1.051285	1.270702
0.080118	0.269442	0.236415	0.059867	0.022084	0.03362	0.252884	0.283703	0.079735
0.040059	0.134721	0.118207	0.029934	0.011042	0.01681	0.126442	0.141852	0.039867

		**0%**	**1%**	**2%**	**3%**	**4%**
**Cv1**	**100% FC**	0.022903	0.022384	0.05944	0.063256	0.132535
	**75% FC**	0.183387	0.10029	0.038933	0.040059	0.134721
	**60% FC**	0.118207	0.029934	0.011042	0.01681	0.126442
**Cv2**	**100% FC**	0.141852	0.039867	0.009868	0.058067	0.05207
	**75% FC**	0.156864	0.178403	0.040896	0.017428	0.215148

**60% FC**	0.023945	0.013059	0.016015	0.070831	0.040576

			75% FC					60% FC
2%	3%	4%	0%	1%	2%	3%	4%	0%
21.05	35.05	25.66	14.455	23.4	17.34	28.15	24.18	16.25
18.2	36.455	22.835	15.615	25.835	19.73	22.69	17.1	14.3
26.17	36.015	26.23	14.65	18.25	20.905	17.73	19.3	13.8
33.695	35.06	22.685	24.3	16.2	14.23	23.95	20.15	14.65
24.77875	35.645	24.3525	17.255	20.92125	18.05125	23.13	20.1825	14.75
6.797515	0.704568	1.854537	4.723969	4.461499	2.947876	4.290548	2.958698	1.059088
3.398757	0.352284	0.927269	2.361985	2.230749	1.473938	2.145274	1.479349	0.529544

		75% FC						60% FC
2%	3%	4%	0%	1%	2%	3%	4%	0%
4.68	5.015	4.305	0.875	3.67	2.17	3.26	3.155	1.64
5.385	7.15	5.425	1.97	3.36	2.96	2.15	1.995	0.96
6.56	5.1	4.155	1.115	2.04	2.98	2.79	2.72	1.045
6.16	6.315	3.455	3.04	1.405	1.41	3.19	2.655	1.365
5.69625	5.895	4.335	1.75	2.61875	2.38	2.8475	2.63125	1.2525
0.834819	1.025971	0.815639	0.980026	1.074355	0.748643	0.50901	0.478737	0.311676

0.41741	0.512986	0.407819	0.490013	0.537178	0.374322	0.254505	0.239369	0.155838

			75% FC					60% FC
2%	3%	4%	0%	1%	2%	3%	4%	0%
1.322171	1.275308	1.234208	0.664896	1.246448	1.264548	1.241145	1.469258	0.196854
1.328797	1.194897	1.019868	1.063043	0.679265	1.232406	1.165243	1.369458	0.267774
1.326021	1.12425	1.170615	0.44625	0.641057	1.127143	1.171295	0.526485	0.258649
1.286567	1.001392	1.036377	1.08931	1.305846	1.094083	1.203262	0.96256	0.168953
1.315889	1.148962	1.115267	0.815875	0.968154	1.179545	1.195236	1.08194	0.223057
0.019736	0.116134	0.104139	0.313728	0.356806	0.081792	0.034856	0.430296	0.047889
0.009868	0.058067	0.05207	0.156864	0.178403	0.040896	0.017428	0.215148	0.023945

1%	2%	3%	4%
12.45	14.15	16.3	12.45
14.75	13.15	14.85	11.95
15.225	15.06	13.45	11.32
12.45	15.28	14.2	15.9
13.71875	14.41	14.7	12.905
1.477805	0.972043	1.210372	2.049496
0.738902	0.486021	0.605186	1.024748

1%	2%	3%	4%
0.575	0.85	1.26	2.13
1.525	1.456	1.415	0.675
1.47	1.395	1.56	2.16
1.725	1.455	1.56	0.89
1.32375	1.289	1.44875	1.46375
0.511051	0.294053	0.1432	0.791616

0.255525	0.147027	0.0716	0.395808

1%	2%	3%	4%
0.296458	0.268952	0.333542	0.52286
0.35552	0.234569	0.235678	0.40148
0.308599	0.312655	0.569245	0.326848
0.308599	0.267661	0.336216	0.401458
0.317294	0.270959	0.36867	0.413161
0.026119	0.03203	0.141662	0.081153
0.013059	0.016015	0.070831	0.040576
